# Effect of Yang-Style Tai Chi on Gait Parameters and Musculoskeletal Flexibility in Healthy Chinese Older Women

**DOI:** 10.3390/sports5030052

**Published:** 2017-07-17

**Authors:** Liye Zou, Chaoyi Wang, Zuguo Tian, Huiru Wang, Yankai Shu

**Affiliations:** 1 Research Institute of Sports Science, Hunan University, Changsha 410089, China; liyezou123@gmail.comcom (L.Z.); thomeland@hnu.edu.cn (Z.T.); 2 College of Physical Education, Jilin University, Changchun 130022, China; chaoyiw@gmail.com; 3 Department of Physical Education, Shanghai Jiaotong University, Shanghai 200240, China; wanghr@sjtu.edu.cn; 4 Department of Sports Science, Jishou University, Jishou 416000, China

**Keywords:** Tai chi, gait parameters, older female

## Abstract

The purpose of the present study was to examine the effect of Yang-style Tai chi (TC) on gait parameters and musculoskeletal flexibility in healthy Chinese female adults. Sixty-six female adults aged >65 years were randomly assigned to either an experimental group (67.9 ± 3.2 years of age) receiving three 90-min simplified 24-form TC sessions for eight weeks, or a control group (67.4 ± 2.9 years of age) who maintained their daily lifestyles. All study participants were instructed to perform a selected pace walking for recording gait parameters (stride length, gait speed, swing cycle time, stance phase, and double support times) at both baseline and after the experiment. Low-limb flexibility and range of motion at specific musculoskeletal regions (hip flexion, hip extension, and plantar flexion, as well as anterior and lateral pelvic tilts, pelvic rotation, and joint range of motion (hip, knee, and ankle)) were also assessed in the present study. Multiple separate 2 × 2 Factorial Analysis of Variance (ANOVA) with repeated measures were used to examine the effects of TC on the abovementioned outcomes between baseline and posttest in the two groups. When compared to those in the control group, older female adults who experienced the 8-week Tai chi intervention demonstrated significant improvements in most of the outcome measures. More specifically, positive changes in the TC group were found, including gait parameter (*p* < 0.001 for all; stride length (1.12 to 1.24, +8.6%), gait speed (1.06 to 1.21, +13.9%), stance phase (66.3 to 61.8, −5.5%), swing phase (33.7 to 38.4, +10.1%), double support time (0.33 to 0.26, −21.1%)), flexibility-related outcomes (hip flexion (90.0 to 91.9, 22.6%, *p* < 0.0001), single hip flexor (6.0 to 2.0, −61.5%, *p* = 0.0386), and plantar flexion (41.6 to 49.7, +17.5%, *p* < 0.0001)), and range of motion (anterior pelvic tilt (9.5 to 6.2, −34.7%, *p* < 0.0001), lateral pelvic tilt (6.6 to 8.3, +23.8%, *p* = 0.0102), pelvic rotation (10.3 to 14.7, 28.2%, *p* < 0.0001), hip range of motion (29.8 to 32.9, +13.5%, *p* = 0.001), and ankle range of motion (28.0 to 32.6, +11.1%, *p* < 0.0001)). The present study supports the notion that the practice of TC has a positive effect on healthy older female adults in improving gait parameters and flexibility, counteracting the normal functional degeneration due to age.

## 1. Introduction

A rapidly increasing population of people aged >65 years is evident in China. In 2013, it was estimated that roughly 125 million people fall under the category of older adults, representing more than 9% of the entire Chinese population. This growing trend will continue and the estimated number of this age group may possibly reach 232 million and 331 million by the years 2030 and 2050, respectively [1,2]. Unfortunately, musculoskeletal function degeneration (e.g., leg power, agility, strength, and flexibility) is commonly observed in this age group and these age-related functional impairments are commonly recognized as critical indicators of the health status of older adults [3,4]. Musculoskeletal system degeneration resulting in an impaired gait pattern is associated with the limited ability of performing activities of daily living (e.g., climbing up and down stairs, kneeling, stooping, overhead or forward reaching), loss of independence, and reduced quality of life [5,6]. Age-related gait impairments are also reported to be correlated with increased morbidity and mortality [7].

Age-related gait impairments represent a devastating public health issue that has attracted attention from researchers worldwide. Researchers have devoted significant amounts of time and energy in searching for optimal exercise interventions to improve gait performance in older adults. Lopopolo et al. [8] conducted a systematic review and concluded that therapeutic exercises (e.g., strength training, stretching training, endurance training, aerobic training) are beneficial for improving gait parameters in older adults only if the high intensity and high dosage in the exercise interventions are evident. Tai chi is a traditional Chinese health-promoting exercise, characterized by slowness, muscle stretching and relaxation, steady rhythm, breathing control, mental focus, and body weight shifting [9,10,11,12]. The majority of previous studies have examined the efficacy of Tai chi for healthy older adults, concentrating on motor function-related outcomes (e.g., flexibility, strength, mobility, leg power, and balance) rather than gait-related parameters [13,14,15]. When researchers have examined the efficacy of Tai chi on gait performance, these studies have focused on special populations, including people with neurological disorders [16,17,18,19] and older people with knee osteoarthritis [20,21]. Because Tai chi has been shown to be a low-to-moderate intensity exercise [22,23,24], it remains unclear whether Tai chi would improve gait performance in the healthy population. Up to now, Tai chi has rarely been investigated regarding its effect on gait parameters, hip flexibility, and joint range of motion among healthy older Chinese women only. Therefore, we were interested in examining the efficacy of Tai chi on the abovementioned parameters in this population. 

## 2. Methods

### 2.1. Study Design

In the present study, we designed a randomized controlled trial to investigate the influence of an 8-week Tai chi course on gait parameters in healthy Chinese older adults. We selected the 8-week training program based on the participants’ availability. The study protocol was approved by the Institutional Review Board (IRB) of Jishou University. Prior to the beginning of the experimental implementation, participants who met the predetermined eligible criteria were required to sign a written consent form. The primary and secondary outcomes were measured at baseline and week 8. 

#### 2.1.1. Study Participants

An e-advertisement was utilized to target the potential study population in a local community center in northeast China. A total of 75 volunteers contacted the primary author and wanted to participate in the present study. Participants were included if they: (1) were 65 years old or above; (2) were able to walk independently; (3) were able to comply with the study protocol; (4) were not currently enrolled in any other behavioral intervention program. Participants were excluded if they: (1) reported any chronic diseases (e.g., knee osteoarthritis, cardiopulmonary disease, low back pain) limiting their physical functions; (2) had actively been participating in a supervised exercising program in the previous three months.

#### 2.1.2. Randomization and Intervention Program

Participants who met the eligibility criteria were arranged for a baseline assessment. Once the baseline assessments were completed, the details of the eligible participants were first entered into Excel and each participant was given a code number ranging from 1 to 54, administered by a graduate student who was blinded to the study protocol. A random sequence generator (retrieved from https://www.random.org/sequences/) was utilized in order to randomly assign 54 eligible participants into either the Tai chi or the control group according to a ratio of 1:1. Participants in the experimental group performed three 90-min simplified 24-short Yang-style Tai chi sessions weekly for 8 weeks, instructed by a health-Qigong master. The intervention protocol consisted of 5 min of warm up (stretching), 5 min of cool-down exercises (self-massage on their own body), and 80 min of Tai chi practice. Participants in the control group signed a consent form and agreed to maintain their daily lifestyle during the intervention period. We used WeChat to communicate and monitor their daily lifestyle.

#### 2.1.3. Adverse Event

We created a worksheet for the eligible participants to record their own adverse events that took place during the intervention period. Such events included, but were not limited to, fall-related fracture, musculoskeletal injuries, or required hospitalization. If a participant experienced any of the abovementioned adverse events, she would be immediately told to withdraw from the present study and see her medical doctors. However, participants who experienced muscle-related soreness, pain, or discomfort three days after Tai chi training across the 8-week intervention period were not recognized as having adverse events. 

### 2.2. Measurement

#### 2.2.1. Physical Activity Level

Prior to the beginning of the experimental implementation, the Baecke Questionnaire was used to evaluate habitual physical activity (HPA) in the previous 12 months [25,26]. The HPA consisted of 16 questions within three dimensions (eight questions for occupational physical activities, OPA; four questions for sports, SP; and four questions for leisure and locomotion physical activities, LLPA). Given that all participants were retired and aged 65 years or older, OPA and SP were excluded from the present study. Because score for each question ranged from one to five, a total of LLPA (in this case, the final score for HPA is equivalent to LLPA) was calculated, with a higher score indicating a better physical activity level. 

#### 2.2.2. Primary Outcomes

Each participant was asked to carry out barefoot walking along a 12-m standardized grid walkway mat at a self-selected speed for two trials. The best performance of the two trials was selected for further data analysis. Three-dimensional kinematic data on walking were recorded using a four-camera infrared motion analysis system (JVC, GR-D250, sampling frequency 60 Hz, Vicon; Oxford Metrics, Oxford, UK). Three skeletal regions were selected for targeting anatomical landmarks of markers: (1) hip (anterior superior iliac crest, greater trochanter); (2) knee (lateral femoral epicondyle); (3) ankle (heel, lateral malleolus, and head of fifth metatarsal). The markers at specific anatomical landmarks were visible in the video images, which were automatically digitized, and the data were converted into three-dimensional data from two-dimensional data (Simi Motion, version 6.1, Los Angeles, CA, USA). The kinematic data in the lower limbs included spatiotemporal parameters (cycle time, stance phase, double support time, swing phase, stride length, and gait speed) and segment-joint angles (anterior pelvic tilt, lateral pelvic tilt, pelvic rotation, and range of motion (ROM) at hip, knee, and ankle).

#### 2.2.3. Secondary Outcome Measures

The modified Thomas Test (MTT) was used to measure flexibility for the hip (iliopsoas and quadriceps) and the flexor of the foot (plantar flexor) [27]. An assessor specializing in athletic training who was blinded to the study protocol performed the MTT as follows. (1) Each participant was instructed to lay down on the flat table in a supine position (lumbar spine was flat on the table and a posterior tilt of the pelvis was avoided); (2) the participant flexed both knees and brought her knees to the chest as close as she possibly can; (3) the participant was instructed to hold one leg and then slowly drop her opposite leg (test leg) to see if the leg made it to the table. If a full extension was achieved with the test leg while the contralateral hip was held in maximal flexion, the angle of hip flexion (reflecting the length of iliopsoas) was measured using a goniometer, ranging from zero to 120 degrees (if a full extension was not achieved, it indicated iliopsoas tightness; if she was able to achieve full hip extension, but 75 degrees of knee flexion was noted, then it indicated rectus femoris tightness). When the test leg started to deviate from the flat table, an angle (single hip flexor) was present relative to the contralateral flexed leg. The angle for plantar flexion was also measured using a goniometer while the participant flexed her ankle joint (the toe and underside of the foot rotated downwards; details of the testing procedure can be retrieved from http://www.topendsports.com/testing/tests/thomas-test.htm). Two trials for each test were conducted, and the average value of both sides were considered as the final value for data analysis. 

#### 2.2.4. Statistical Analysis

All statistical analyses were carried out with IBM SPSS version 23.0 (SPSS Inc., Chicago, IL, USA). The level of significance for the present study was set at 0.05. Prior to the beginning of examining the main interest of the present study, preliminary analyses were conducted to examine the demographic variables (e.g., age, Bone mineral density (BMI), and physical activity level). Means and standard deviations were used to summarize continuous data. Differences at baseline in the demographic information between the two groups were compared using a *t*-test for continuous data. Multiple separate 2 × 2 Factorial Analysis of Variance (ANOVA) with repeated measure were used to examine: (1) if there was significant improvement found in outcome measures between baseline and week 8 in both experimental and control groups; (2) whether participants receiving 8-week Tai chi training would demonstrate significantly better scores on outcome measures than those in the control group. A two-tailed *p* value of 0.05 was set to determine statistical significance. Between-group differences with 95% confidence intervals were used to demonstrate the results of the study. 

## 3. Results

### 3.1. Study Participants

**Figure 1:**
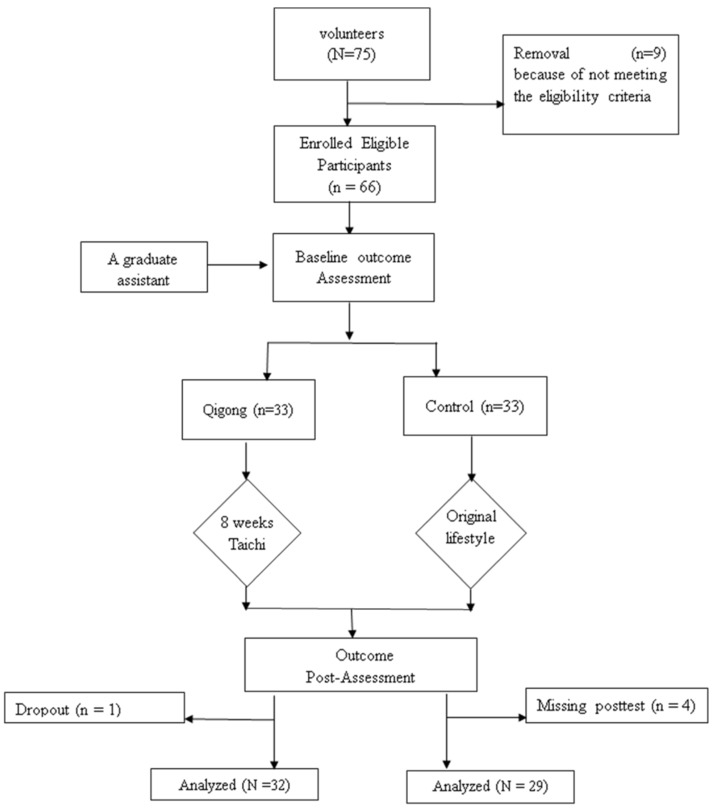
Flowchart showing the process of participant recruitment and experiment implementation.

According to the abovementioned eligibility criteria, nine volunteers were excluded due to schedule conflict (*n* = 4), being physically active in the past three months (*n* = 3), or low back pain (*n* = 2). A final total of 61 participants was included in the present study because three participants were not considered for data analysis: (1) one participant withdrew during the intervention period without reporting the reason for discontinuing the TC training; (2) in the control group, four participants missed the posttest measurements (Figure 1 shows the process of participant recruitment and experimental implementation). The present study indicates high compliance with TC training (the mean attendance rate was at 93%, with an attrition rate of 3% between baseline and week 8). With regard to the characteristics of the study participants at baseline, the average ages were 67.9 ± 3.2 years in the TC group and 67.4 ± 2.9 years in the control group, respectively. All participants were female and retired faculty members with a Master’s degree or above. BMI level in the TC group was 25.1 ± 4.2, while it was 25.7 ± 3.0 in the control group. Their physical activity level as measured by the Baecke Questionnaire was 6.15 ± 0.5 in the TC group and 6.1 ± 0.3 in the control group. None of the baseline outcome measures between the two groups were significantly different (*p* > 0.05).

### 3.2. Effects of an 8-Week Tai Chi Training Program on Gait Parameters

Table 1 shows the within-group and between-group differences of gait parameters for the two groups, including stride length, gait speed, cycle time, stance phase, swing phase, and double support time. At baseline, no significant difference was observed on all the outcome measures between the TC and control groups (*p* > 0.05). At week 8, significant improvements in the majority of outcome measures (*p* < 0.05 except for cycle time) were only found in the TC group, not the control group. More specifically, the TC group experienced positive changes in stride length (1.12 to 1.24, +8.6%), gait speed (1.06 to 1.21, +13.9%), stance phase (66.3 to 61.8, −5.5%), swing phase (33.7 to 38.4, +10.1%), and double support time (0.33 to 0.26, −21.1%). For the stride length, gait speed, and swing phase, a higher positive value of percentage change indicates better performance. For the cycle time, stance phase, and double support time, a higher negative value of percentage change indicates better performance. 

### 3.3. Effects of an 8-Week Tai Chi Training Program on Flexibility

Table 2 shows the within-group and between-group differences of flexibility-related outcomes for the Tai chi and control groups, including hip flexion (iliopsoas), single hip flexor (rectus femoris), and plantar flexion (plantar flexor) as measured by the Thomas Test. At baseline, no significant difference was evident on all the outcome measures between the TC and control groups (*p* > 0.05). At week 8, significant improvements in all the outcome measures (*p* < 0.05 for all outcome measures) were only found in the TC group, not the control group. More specifically, the TC group experienced positive changes on hip flexion (iliopsoas) (90.0 to 91.9, 22.6%), single hip flexor (rectus femoris) (6.0 to 2.0, −61.5%), and plantar flexion (plantar flexor) (41.6 to 49.7, +17.5%). For the hip flexion (iliopsoas) and plantar flexion (plantar flexor), a higher positive value of percentage change indicates better performance. For the single hip flexor (rectus femoris), a higher negative value of percentage change indicates better performance. 

### 3.4. Effects of an 8-Week Tai Chi Training Program on Joint Range of Motion

Table 3 shows the within-group and between-group differences of joint range of motion for the two groups, including anterior pelvic tilt (APT), lateral pelvic tilt (LPT), pelvic rotation (PRO), hip range of motion (HROM), knee range of motion (KROM), and ankle range of motion (AROM). At baseline, no significant difference was observed on all the outcome measures between the TC and control groups (*p* > 0.05). At week 8, significant improvements in the majority of outcome measures (*p* < 0.05, except for KROM) were only found in the TC group, not the control group. More specifically, the TC group experienced positive changes on ALP (9.5 to 6.2, −34.7%), LPT (6.6 to 8.3, +23.8%), PRO (10.3 to 14.7, 28.2%), HROM (29.8 to 32.9, +13.5%), and AROM (28.0 to 32.6, +11.1%). For LPT, HROM, KROM, and AROM, a higher positive value of percentage change indicates better performance. For APT, a higher negative value of percentage change indicates better performance. 

## 4. Discussion

The purpose of the present study was to determine the changes in gait parameters, lower-limb muscle flexibility, and joint range of motion in a group of female participants aged >65 years who participated in an 8-week Tai chi (TC) training program and those who maintained their original lifestyle (control group, CG). The main findings are as follow: (1) of the gait parameters, three (stride length, gait speed, and swing phase) were greater and two (stance phase and double support times) became lower for the TC group after the 8-week intervention period, but not for the CG; (2) the flexibility of the lower-limb muscles (hip flexors (iliopsoas), knee extensors (rectus femoris), and plantar flexors (plantar flexor)) in the TC group were all improved, and; (3) the lower-limb ROMs were improved comprehensively, as indicated by improved anterior and lateral pelvic tilts, pelvic rotation, hip ROM, and ankle ROM. 

After the 8-week intervention period, decreased double support time in the stance phase was observed in the TC group, but not CG, resulting in more percentage of cycle time devoted to the swing phase rather than the stance phase for the TC group. Compared to a single support, double support provides a larger area of stable surface. Older people typically have a longer double support time per gait cycle to maintain gait stability and may reduce the risk of falls as compared to younger adults [28]. Older people tend to improve gait stability by reducing the swing phase time and increasing the gait structure of stance phase time, which is consistent with what we observed in the CG and TC group at the baseline. According to Murray [29], the normal stance phase accounts for 60% of the cycle time and the normal swing phase accounts for 40% of the cycle time. The ratio of stance phase (61.8 ± 2.6%) to swing phase (38.4 ± 1.9%) of older participants in the TC group is closer to the ratio of healthy younger subjects, which indicates a return of normal gait patterns for older people in the TC group after the 8-week TC training. These positive findings may be attributed to the feature of Tai chi movements, particularly Distinguish Substantial and Insubstantial Leg (Fen Qing Xu Shi) movement. TC requires practitioners to achieve agility and smoothness in shifting weight from one leg to another. Substantial (shi) literally means “solid,” implying firmness and stability, while insubstantial (xu) literally means “empty,” implying the ability to change [30,31]. This guideline results in shifting a majority of the body weight to the substantial leg and promotes more time spent in the single-leg support phase at each TC walking cycle. Thus, older participants may become more confident while walking in the single support phase after the 8-week TC training. 

The results indicate that, after the 8-week intervention period, older female participants in the TC group demonstrated significant improvement on gait speed (from 1.06 to 1.21 m/s) and stride length (from 1.12 to 1.24 m), whereas the gait speed and stride length of the CG remained the same over the 8 weeks. It is worth mentioning that the value of the gait speed of the older participants at the baseline in the present study were consistent with those reported in a previous study [28]. Wayne et al. [31] reported that the gait speed for single task not dual task was also 1.12 m/s. The gait speeds for healthy adults have been shown to range from 1.05 to 1.43 m/s [32,33]. Reductions in gait speed, stride length, ranges of motion, and force momentum in hip and knee joints are common in older adults [34,35,36]. In particular, the reduction in freely selected gait speed is an indicator of risk of falls [37]. Previous studies have shown that the kinematic parameters of the knee and ankle joints have a positive relationship with gait speed [38,39,40]. In particular, Kwon, Son, and Lee [41] found that between the pre-swing and the mid-swing, the peak values of the flexion and the external rotation of the knee joint and the peak values of the plantar-flexion of the ankle joint significantly increased with the increase of gait speed. In the present study, although we failed to find an improvement in knee ROM, the increases in hip and ankle ROM as well as the increases in iliopsoas, knee extensors, and plantar flexor flexibility in the TC group may provide a perquisite for the older female participants to generate a greater range of motion and joint power to increase gait speed and stride length. 

The study findings of the present study suggest that the 8-week TC training can increase the flexibility of the hip, the surrounding tissues of the pelvis, and the ankle. At the end of the training, the hip flexibility reached a relatively high degree of 91.9 ± 8.0, while rectus femoris and plantar flexion flexibility also improved significantly. However, no significant changes in hip and ankle flexibility were observed in the CG. A study by Kerrigan et al. [39] showed that individuals who reduce hip joint movement are more likely to fall. A large number of studies have shown that strength training programs have a beneficial impact on gait efficacy [42,43], including slowing down the decline in gait function due to age. However, with regard to hip flexibility, a few studies have shown that the stretching training program is better than the strength training program to retard the normal, age-related contraction of the muscle groups of the pelvis and hip, like the gluteal and rotator [44,45]. The results of our study indicate that the 8-week TC training could improve the flexibility at the hip, knee, and ankle joints. This probably is associated with the “Loosen Waist and Hips (Song Yao Song Kua)” guideline of TC. From a martial arts standpoint (Tai chi is a traditional Chinese martial arts), when the waist and hips are loose, the power generated by the lower limbs (e.g., ankle, knee, hip) can easily be transmitted to the arms. To adhere to this principle, the upper body must be upright and the stance must be comfortable, which may be effective in improving the flexibility of the hip and ankle.

In conclusion, an 8-week Tai chi training may change gait parameters, lower-limb flexibility, and ROM in joints (ankle, knee, hip) to decrease risk of falls in older people. Kerrigan et al. [42] found that decreased stride length is correlated with increased risk of falls. More specifically, the researchers emphasized that restricted hip muscle resulted from the reduction in hip extension in combination with an anterior tilt of the pelvis is a primary reason for a decrease in stride length and walking speed in older people who often fall [42]. The larger the degree of lateral pelvic tilt and pelvic rotation, the more stable the heel of the swinging leg that supports the lower limbs [46]. In addition, a study by Chiacchiero et al. [47] indicated that decreased passive lower extremity ROM and flexibility may contribute to falls in the older people. In the present study, the increase in hip flexibility (iliopsoas and rectus femoris), anterior pelvis tilt, and anterior pelvis rotation of the TC group indicate that 8-week TC training may contribute to an increase in the stride length and gait speed, thus reducing the probability of falls in older people. 

### Study Limitation

This study examines the effect of Tai chi on gait parameters in older people, mainly based on kinematic data. Future studies may consider providing kinetic data in order to reach a more comprehensive conclusion. In the present study, the gait parameter data were extracted based on the better performance of two trials, instead of the average score. According to Maynard et al. [48], a minimum of three gait cycles should be averaged to overcome the effects of stride-to-stride variability. Future studies should consider collecting the gait parameter data based on three or more trials to give a more reliable result. The present study only tested the flexibility of three lower-extremity muscles; hip flexion (iliopsoas), single hip flexor (rectus fermoris), and plantar flexion. More flexibility tests in lower-limb muscles (e.g., iliotibial band, hamstrings, and gastrocnemius) are needed in order to more specifically know whether other muscle flexibility could be improved by TC training. Future research is also required to expand the findings of the lower limb ROM and flexibility in the present study by examining the other joints’ mobility (e.g., shoulder and elbow); as Tai chi emphasizes, “Once the waist moves, the other joints in the body are affected.” The functional level in ambulation and risk of fall best reflects the physical capability in their real lives, which should also be evaluated by investigators of future studies. Considering that the degeneration of the musculoskeletal system and aerobic metabolism resulting in impaired gait pattern and walking endurance are associated with the limited capability of performing activities of daily living, future studies should examine whether Tai chi has a positive effect on related physical fitness (e.g., muscle strength, cardiorespiratory endurance, and muscular endurance). The present study only contained a control group compared to the Tai chi group; if future studies could add a placebo control group (stretching program) in a randomized controlled trial, it would be more convincing to explain a cause-and-effect relationship between the Tai chi training program and health-related parameters. 

## 5. Conclusions

Yang-style Tai chi is effective in improving gait parameters and musculoskeletal flexibility in healthy Chinese older adults. It should be thought of as an alternative method for older female adults to slow down the normal decline in gait performance and physical flexibility. Future studies should run a similar randomized controlled trial with a large sample size to confirm the beneficial effects of Yang-style Tai chi on gait parameters and musculoskeletal flexibility in different populations such as healthy male older adults, people with Parkinson’s disease, and people with peripheral neuropathy. 

## Figures and Tables

**Table 1:** Within-group and between-group comparisons for gait parameters at baseline and week 8 (*n* = 61) using repeated measures ANOVA.

Outcome Measure	Within-Group Effects				Between-Group Effects
	Baseline	Week 8	*p*	Baseline—Week 8	*p*
	Mean ± SD	Mean ± SD		Percentage Change	
Stride length (m)					<0.0001
TC (*n* = 32)	1.12 ± 0.08	1.24 ± 0.09	<0.05	+8.6	
CG (*n* = 29)	1.12 ± 0.05	1.12 ± 0.13	>0.05	0.2	
Gait speed (m/s)					0.0017
TC (*n* = 32)	1.06 ± 0.17	1.21 ± 0.23	<0.05	+13.9	
CG (*n* = 29)	1.06 ± 0.07	1.06 ± 0.09	>0.05	+0.4	
Cycle time (sec)					0.6783
TC (*n* = 32)	1.09 ± 0.12	1.08 ± 0.09	>0.05	−1.0	
CG (*n* = 29)	1.08 ± 0.05	1.08 ± 0.09	>0.05	+0.7	
Stance phase (%)					<0.0001
TC (*n* = 32)	66.3 ± 4.1	61.8 ± 2.6	<0.05	−5.5	
CG (*n* = 29)	66.9 ± 4.0	66.0 ± 3.8	>0.05	−1.4	
Swing phase (%)					<0.0001
TC (*n* = 32)	33.7 ± 2.3	38.4 ± 1.9	<0.05	+10.1	
CG (*n* = 29)	33.1 ± 2.0	33.0 ± 2.8	>0.05	−2.8	
Double support time (sec)					<0.0001
TC (*n* = 32)	0.33 ± 0.05	0.26 ± 0.05	<0.05	−21.1	
CG (*n* = 29)	0.33 ± 0.02	0.33 ± 0.01	>0.05	−0.7	

Note: TC = Tai chi Quan; CG = control group.

**Table 2:** Within-group and between-group comparisons for flexibility at baseline and week 8 (*n* = 61) using repeated measures ANOVA.

Outcome Measure	Within-Group Effects				Between-Group Effects
	Baseline	Week 8	*p*	Baseline—Week 8	*p*
	Mean ± SD	Mean ± SD		Percentage Change	
Hip flexion (degree)					< 0.0001
TC (*n* = 32)	70.0 ± 6.8	91.9 ± 8.0	<0.05	+22.6 *	
CG (*n* = 29)	69.5 ± 3.7	69.9 ± 4.5	>0.05	+0.4	
Rectus femoris (degree)					0.0386
TC (*n* = 32)	6.0 ± 2.5	2.0 ± 1.3	<0.05	−61.5 *	
CG (*n* = 29)	5.8 ± 1.6	3.0 ± 2.3	>0.05	−2.0	
Plantar flexion (degree)					<0.0001
TC (*n* = 32)	41.6 ± 5.3	49.7 ± 3.1	<0.05	+17.5 *	
CG (*n* = 29)	42.0 ± 4.2	42.7 ± 7.4	>0.05	+0.9	

Note: TC = Tai chi Quan; CG = control group; Asteroid * indicates (asteroid) significant difference.

**Table 3:** Within-group and between-group comparisons for joint range of motion at baseline and week 8 (*n* = 61) using repeated measures ANOVA.

Outcome Measure	Within-Group Effects				Between-Group Effects
	Baseline	Week 8	*p*	Baseline—Week 8	*p*
	Mean ± SD	Mean ± SD		Percentage Change	
APT (degree)					<0.0001
TC (*n* = 32)	9.5 ± 0.8	6.2 ± 1.8	<0.05	−34.7	
CG (*n* = 29)	9.6 ± 0.9	9.4 ± 2.1	>0.05	−2.5	
LPT (degree)					0.0102
TC (*n* = 32)	6.6 ± 0.7	8.3 ± 2.1	<0.05	+23.8	
CG (*n* = 29)	6.7 ± 2.8	6.7 ± 2.6	>0.05	+0.3	
PRO (degree)					<0.0001
TC (*n* = 32)	10.3 ± 2.0	14.7 ± 2.9	<0.05	+28.2	
CG (*n* = 29)	10.8 ± 6.4	10.7 ± 3.9	>0.05	−1.8	
HROM (degree)					0.0010
TC (*n* = 32)	29.8 ± 4.5	32.9 ± 2.5	<0.05	+13.5	
CG (*n* = 29)	29.9 ± 3.2	29.7 ± 4.5	>0.05	−0.9	
KROM (degree)					0.5245
TC (*n* = 32)	53.2 ± 4.8	54.9 ± 5.7	>0.05	+2.9	
CG (*n* = 29)	53.7 ± 4.0	53.9 ± 6.5	>0.05	+0.4	
AROM (degree)					<0.0001
TC (*n* = 32)	28.0 ± 4.2	32.6 ± 5.1	<0.05	+11.1	
CG (*n* = 29)	27.9 ± 3.6	27.1 ± 2.1	>0.05	−0.2	

Note: TC = Tai chi Quan; CG = control group; APT = anterior pelvic tilt; LPT = lateral pelvic tilt; PRO = pelvic rotation; HROM = hip range of motion; KROM = knee range of motion; AROM = ankle range of motion.
